# Drought-Driven Divergence in Photosynthetic Performance Between Two *Cunninghamia lanceolata* Provenances: Insights from Gas Exchange and Chlorophyll Fluorescence Dynamics

**DOI:** 10.3390/plants14101487

**Published:** 2025-05-15

**Authors:** Xiaofei Gong, Ziyun Wan, Peng Jin, Songheng Jin, Xueqin Li

**Affiliations:** 1 Jiyang College, Zhejiang A&F University, Zhuji 311800, China; gongxf6868@sina.com (X.G.); 2020105012012@stu.zafu.edu.cn (Z.W.); 17857218867@163.com (P.J.); shjin@zafu.edu.cn (S.J.); 2 Suichang County Ecological Forestry Development Center, Lishui 323300, China

**Keywords:** *Cunninghamia lanceolata*, photosynthetic electron transport, PSI/PSII activity, provenance variation, drought adaptation, forestry sustainability

## Abstract

*Cunninghamia lanceolata*, contributing 25% to China’s commercial timber production, faces severe drought threats. However, provenance-specific photosynthetic adaptations remain poorly understood. Here, we compared gas exchange, prompt/delayed fluorescence (PF/DF), and modulated 820-nm reflection (MR) responses of two provenances (JXJJ and FJSM) under different drought treatment times. JXJJ maintained a higher net photosynthetic rate (Pn) and stomatal conductance (Gs) than FJSM under drought stress. The declining rates of F_V_/F_M_, φE_O_, Ψ_O_, δR_O_, PI_ABS_, TR_O_/CS_M_, and ET_O_/CS_M_ were much more rapid in the FJSM than in the JXJJ. An MR kinetics analysis revealed significantly greater PSI impairment in FJSM, evidenced by a 60.2% reduction in P700^+^ re-reduction rate (V_red_) compared to only 44.4% in JXJJ (*p* < 0.05) at 20 d drought treatment. Similarly, DF measurements demonstrated more pronounced PSII energy transfer disruption in FJSM, with the I_2_/I_1_ ratio increasing by 51.3% vs. 43.0% in JXJJ at 20 d drought treatment. These results demonstrate JXJJ’s superior drought resilience through coordinated stomatal and non-stomatal regulation. Our findings provide actionable criteria for selecting drought-tolerant *C. lanceolata* provenances, which is essential for sustainable forestry as the climate changes. This study underscores the significance of photosynthetic activity in how *C. lanceolata* responds to drought and gives insights into boosting drought tolerance in forest species through genetic improvements.

## 1. Introduction

Plants employ physiological strategies to adapt to diverse environmental conditions and withstand unfavorable biotic and abiotic stresses [1]. Environmental stresses, such as drought, salinity, high temperatures, and frost, cause substantial economic losses in agriculture annually [2]. The escalating global population has intensified water resource demands, exacerbating water scarcity [3]. Climate change further worsens drought conditions, constraining water resource availability [4]. Drought stress is a prevalent abiotic stressor globally, adversely affecting plant growth, development, and productivity [5].

*Cunninghamia lanceolata* (*C. lanceolata*), a fast-growing economic tree species in China, holds significant importance. It ranks first among the country’s plantation timber species in terms of both total area and stock volume, contributing about 25% to the nation’s commercial timber production [6]. Thus, it plays a crucial role in carbon sequestration and maintaining ecological balance [7]. China has over 17 million hectares of *C. lanceolata* plantations, constituting 6.1% of the world’s total plantation forests and playing a vital role in the global carbon cycle (https://www.fao.org/forest-resources-assessment/past-assessments/fra-2015/en/, accessed on 7 September 2015). However, adverse weather conditions in southern China, particularly prolonged droughts linked to global warming, have led to the substantial mortality of *C. lanceolata* seedlings [8,9]. Previous research has predominantly focused on the physiological and biochemical bases of drought tolerance in *C. lanceolata*, but investigations into photosynthetic mechanism changes and provenance variations under drought stress remain limited [10].

Provenance variation is pivotal in shaping tree species’ adaptability to environmental stresses [11]. Genetic differences among provenances influence traits like gas exchange and photosynthetic efficiency, which are essential for evaluating plant performance under drought. Photosynthesis, a fundamental plant metabolic process, is highly sensitive to water availability and is significantly impacted under drought conditions [12]. Gas exchange parameters, including net photosynthesis rate (Pn), stomatal conductance (Gs), and intercellular CO_2_ concentration (Ci), offer insights into plant water-use efficiency and photosynthetic capacity [13]. Moreover, signals from prompt chlorophyll a fluorescence (PF) transients (OJIP), delayed chlorophyll a fluorescence (DF), modulated 820-nm reflection (MR), energy conversion efficiencies in photosystems I (PSI) and II (PSII), and cyclic electron flow (CEF) activity are widely used to monitor changes in photosynthetic light reactions [14,15,16]. These parameters provide valuable insights into the functional state of photosynthetic machinery under various environmental conditions [17].

In this study, we selected two provenances of *C. lanceolata* from different latitude regions in China as experimental materials, and assessed the seedlings under drought conditions, measuring OJIP signals, DF, MR, and gas exchange parameters to evaluate their physiological responses and adaptability. The *C. lanceolata* growing in Sanming, Fujian Province (FJSM) is located in the central subtropical climate zone, while the *C. lanceolata* growing in Jiujiang, Jiangxi Province (JXJJ) is located in the northern subtropical climate zone. These two regions are the main areas for *C. lanceolata* production in China. While prior studies have reported on *C. lanceolata*’s response to drought stress, research on physiological differences, gas exchange, and chlorophyll fluorescence responses among different provenances from different latitude regions remains limited. Therefore, analyzing the gas exchange and fluorescence characteristics of two provenances under drought stress can provide a theoretical foundation for selecting and breeding drought-tolerant varieties.

## 2. Results

### 2.1. Soil Moisture Content and Gas Exchange Parameters

**Figure 1:**
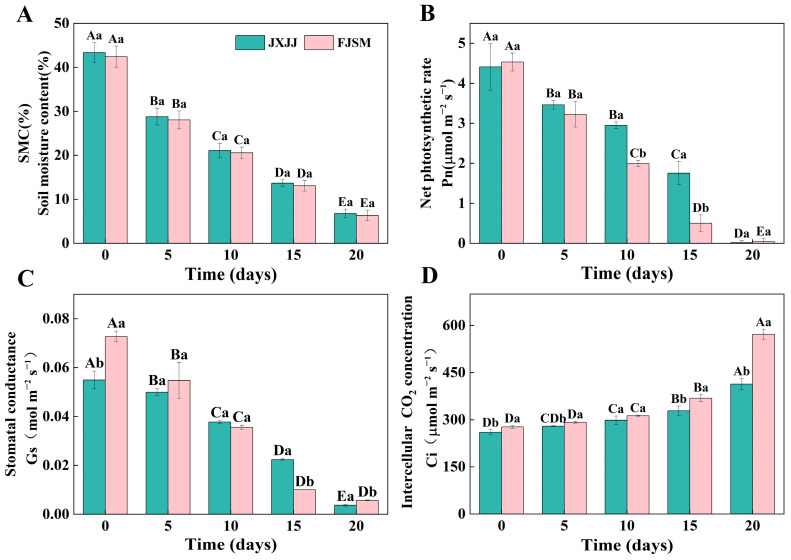
Changes in soil moisture content and gas exchange parameters in *Cunninghamia lanceolata* under drought stress. (**A**–**D**) represent soil moisture content (SMC), net photosynthesis rate (Pn), stomatal conductance (Gs), and intercellular CO_2_ concentration (Ci), respectively. Data are the mean ± SE of six biological replicates. Different lowercase letters indicate significant differences between provenances at the same time (*p* < 0.05), and uppercase letters indicate significant differences across drought durations for the same provenance (*p* < 0.05).

Throughout the drought treatment (0 d, 5 d, 10 d, 15 d, and 20 d), the Soil moisture content (SMC) decreased progressively across both provenances (Figure 1A). At 20 d of drought treatment, SMC decreased by 84% in JXJJ and 85% in FJSM compared to the control. Drought treatment had a noticeable effect on the gas exchange parameters. JXJJ maintained a relatively higher Pn compared to FJSM throughout the treatment period (Figure 1B). After 10 d of drought stress, provenance deviations were evident, with reductions of 33% in JXJJ and 56% in FJSM. The divergence reached its maximum after 15 d drought treatment, with decreases of 60% and 89%, respectively. The Gs of both provenances decreased significantly with increasing drought stress. After 15 d of stress, Gs in JXJJ was significantly higher than in FJSM, with reductions of 60% and 86%, respectively (Figure 1C). Nevertheless, Ci showed an opposite trend. After 20 d of drought stress, Ci in FJSM increased by 106% compared to JXJJ, approximately twice the rate observed in JXJJ (Figure 1D).

### 2.2. Prompt Fluorescence OJIP Transient Analysis

**Figure 2:**
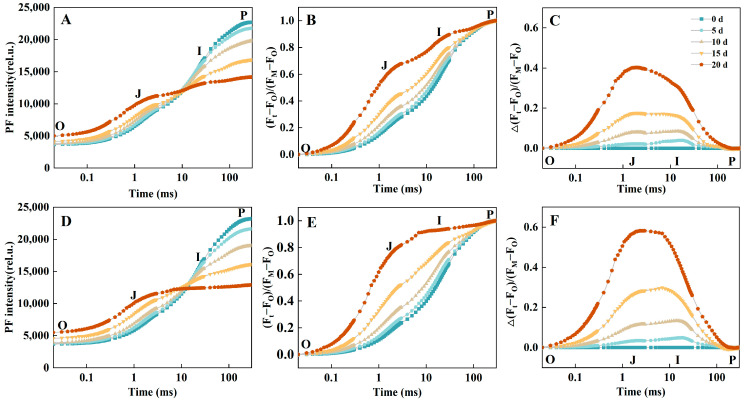
The PF transients of leaves under drought stress. (**A**) Absolute values of JXJJ. (**B**) Normalized transients of JXJJ. (**C**) The ΔVt curves of JXJJ. (**D**) Absolute values of FJSM. (**E**) Normalized transients of FJSM. (**F**) The ΔVt curves of FJSM. The signals were plotted on a logarithmic time scale. Data are the mean of six biological replicates.

As drought stress prolonged, significant differences in OJIP transient curves were observed between the two provenances. JXJJ exhibited relatively stable OJIP curves, particularly in the fluorescence rise at the J, I, and P phases, indicating a mild damage on the PSII functionality. The PF decline in both provenances showed a pattern dependent on the logarithmic time scale, as presented in Figure 2. For JXJJ, F_P_ decreased by 4.3%, 12.9%, 25.8%, and 37.7% after 5, 10, 15, and 20 d, indicating a gradual reduction in PSII activity (Figure 2A). In FJSM, the reductions were more obvious, amounting to 6.9%, 14.4%, 30.5%, and 44.5% over the same drought durations (Figure 2D). This implies that FJSM is more susceptible to drought-induced photochemical stress compared to JXJJ. The normalization of the OJIP curve between the O point (20 μs) and the P point (300 ms) was analyzed on logarithmic time axis (Figure 2B,E), with the ΔV_t_ curve showing a distinct peak at the J step (Figure 2C,F). The J step of the FJSM OJIP transient was notably higher starting from 15 d of drought stress (Figure 2B), while the increase in the J step for the JXJJ OJIP transient became more apparent only after 20 d of drought stress (Figure 2E). The relative contribution of the J step increased under drought, as normalized data showed stable J-step fluorescence but decreased J-I-P amplitudes. Exposed to drought stress, distinct peaks in the ΔV_t_ curves of both provenances were observed at the J step. ΔJ increased to 0.02, 0.08, 0.18, and 0.40 after 5, 10, 15, and 20 d of drought treatment in JXJJ, respectively. In FJSM, ΔJ increased to 0.03, 0.11, 0.27, and 0.58 after 5, 10, 15, and 20 d of drought stress, respectively.

Double-normalization of the OJIP transients between F_O_ and F_J_ or F_O_ and F_K_, followed by subtracting the double-normalized signals of the control group from those of the treatment group, enables visualization of the K-band (0.05 and 2 ms) and the L-band, respectively [18]. A clear positive K-band was observed in both provenances as early as 10 d of drought treatment, with its intensity progressively increasing as drought stress continued (Figure S1A–D). There is no significant difference in the k-band between FJSM and JXJJ. Furthermore, as the drought stress persisted, the amplitude of the L-band gradually rose, attaining the maximum level after 20 d treatment. As illustrated in Figure S1G,H, the L-band in FJSM under 15 d of drought stress reached a level comparable to that of JXJJ after 20 d of drought stress (Figure S1E–H).

### 2.3. JIP-Test

**Figure 3:**
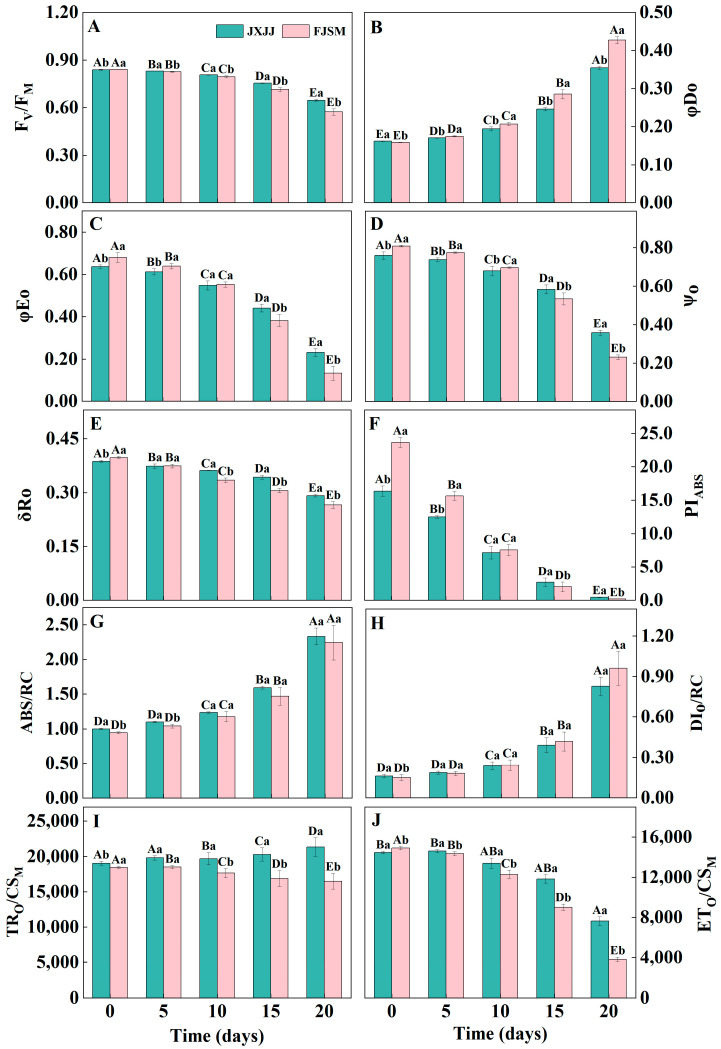
Parameters derived from PF transients of leaves in *Cunninghamia lanceolata* under drought stress. (**A**) The maximum photochemical efficiency of PSII (F_V_/F_M_). (**B**) The quantum efficiency of energy dissipation (φD_O_). (**C**) The quantum yield for electron transport (φE_O_). (**D**) The probability that an electron moves further than Q_A_^−^ (Ψ_O_). (**E**) The efficiency of an electron beyond Q_A_^−^ that reduced PSI acceptors (δR_O_). (**F**) The performance index on an absorption basis (PI_ABS_). (**G**) The absorption of antenna chlorophyll per PSII reaction center (ABS/RC). (**H**) Dissipation per Reaction Center (DI_O_/RC). (**I**) The rate of primary photochemical capture per unit cross-sectional area (TR_O_/CS_M_). (**J**) Electron transport rate per unit area (ET_O_/CS_M_). Data are the mean ± SE of six biological replicates. Different lowercase letters indicate significant differences between provenances at the same time (*p* < 0.05), and uppercase letters indicate significant differences across drought durations for the same provenance (*p* < 0.05).

To quantitatively analyze the functional and structural changes in photosynthetic apparatus, a variety of parameters were developed from OJIP transients using the JIP-test. F_V_/F_M_, φE_O_, ψ_O_, δR_O_, PI_ABS_, and ET_O_/CS_M_ were gradually decreased with increasing drought treatment time, and the decrease in these parameters of FJSM is greater than that of JXJJ (Figure 3A,C–F,J). φD_O_, ABS/RC, and DI_O_/RC were gradually increased with increasing drought treatment time; the increase in φD_O_ of FJSM was greater than that of JXJJ (Figure 3B,G,H). There was no significant difference in ABS/RC and DI_O_/RC between JXJJ and FJSM (Figure 3G,H). TR_O_/CS_M_ was gradually increased in JXJJ, while it was gradually decreased in FJSM with the increase of drought time (Figure 3I). Evidently, prolonged drought stress clearly led to the inactivation of some active RCs of PSII, hindering electron transfer in photosynthesis. This greatly reduced the maximum photochemical quantum yield of PSII, with the suppression being more pronounced in FJSM.

### 2.4. MR/MR_0_ Transient Analysis

**Figure 4:**
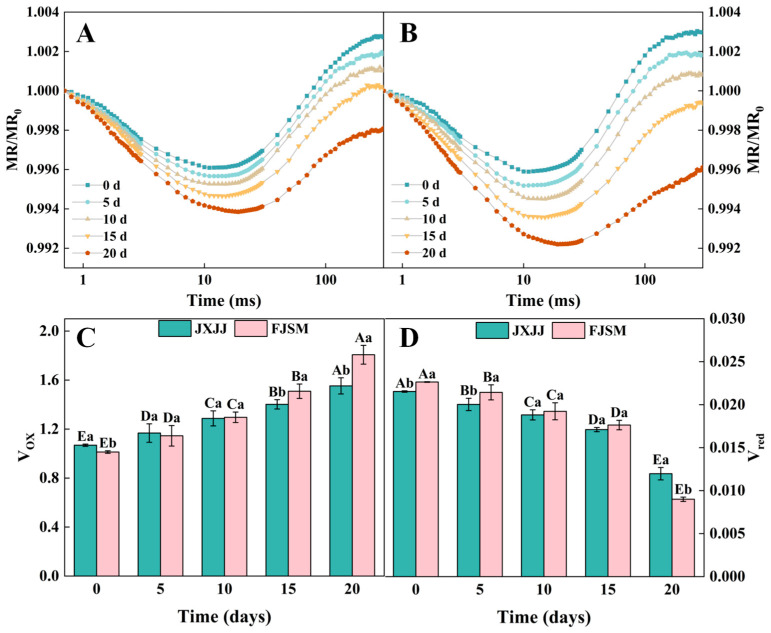
The MR/MR_0_ kinetics of leaves in *Cunninghamia lanceolata* under drought stress. (**A**) The MR/MR_0_ curves of JXJJ. (**B**) The MR/MR_0_ curves of FJSM. (**C**) The absolute value of the maximum decrease in the slope (V_ox_). (**D**) The absolute value of the initial rate of P700^+^ re-reduction (V_red_). Data are the mean (**A**,**B**) or mean ± SE (**C**,**D**) of six biological replicates. Different lowercase letters indicate significant differences between provenances at the same time (*p* < 0.05), and uppercase letters indicate significant differences across drought durations for the same provenance (*p* < 0.05).

Simultaneous measurements of 820 nm light reflection by leaves were conducted to evaluate the redox states of plastocyanin (PC) and P700. The accumulation of P700^+^ and PC^+^ increased absorbance at 820 nm, thereby reducing the MR/MR_0_ ratio (Figure 4). In the absence of drought stress, the normalized MR/MR_O_ of both JXJJ and FJSM initially decreased and then gradually increased (Figure 4A,B). In response to drought treatment, the dynamics of MR/MR_0_ in two provenances exhibited discernible alterations. With increasing drought stress duration, the lowest point in the MR/MR_0_ dynamics for both provenances appeared later. In addition, as drought stress continued, significant changes were observed in the absolute value of the maximum decrease in the slope (V_ox_) and the initial rate of P700^+^ re-reduction (V_red_) of both provenances (Figure 4C,D). The variations in V_ox_ were more considerable in FJSM, highlighting that, compared to JXJJ, the PSI activity in FJSM was more severely impaired. The MR kinetics analysis revealed significantly more severe PSI impairment in FJSM after 20 d of drought stress, with a 60.2% reduction in V_red_ compared to only 44.4% in JXJJ.

### 2.5. DF Induction and Decay Transient Analysis

**Figure 5:**
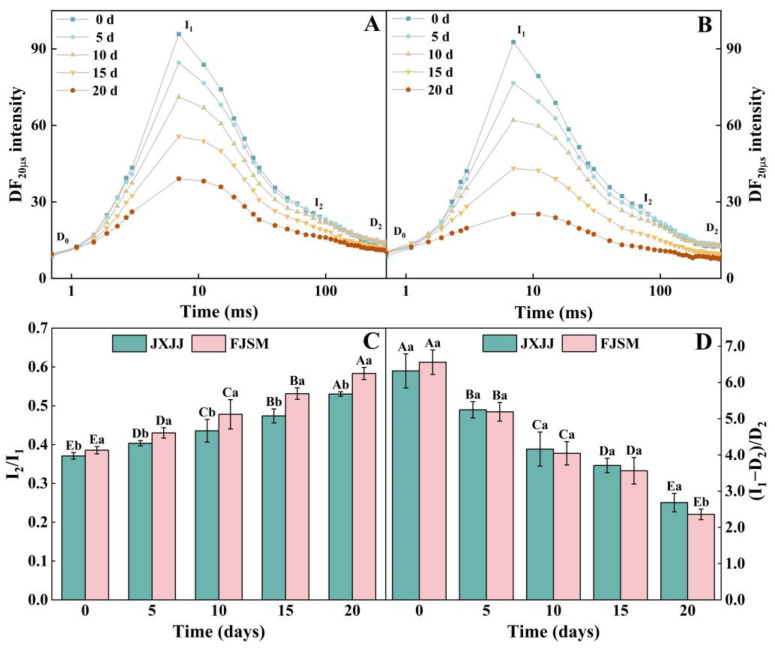
DF induction and decay kinetics of leaves in *Cunninghamia lanceolata* under drought stress. (**A**) The DF curves of JXJJ. (**B**) The DF curves of FJSM. (**C**) The I_2_/I_1_ ratio (**D**) The (I_1_ − D_2_)/D_2_ ratio. I_1_, maximum value. I_2_, second peak value. D_2_, minimum value. Data are the mean (**A**,**B**) or mean ± SE (**C**,**D**) of six biological replicates. Different lowercase letters indicate significant differences between provenances at the same time (*p* < 0.05), and uppercase letters indicate significant differences across drought durations for the same provenance (*p* < 0.05).

DF curves were mapped with fluorescence signals at 20 µs to clearly and intuitively show the shape changes of these curves on a time scale (Figure 5). During the drought treatment, the DF curves of JXJJ and FJSM altered in both amplitude and shape. As the drought stress intensified, DF amplitude progressively declined, with a more noticeable reduction observed at the I_1_ point compared to the I_2_ point (Figure 5A,B). This implied that JXJJ maintains better PSII function under drought conditions compared to FJSM, which might be more prone to damage from prolonged drought stress. Additionally, the I_2_/I_1_ ratio consistently increased, while the (I_1_ − D_2_)/D_2_ ratio continuously decreased under prolonged drought conditions (Figure 5C,D). Delayed fluorescence analysis revealed a significantly greater increase in the I_2_/I_1_ ratio in FJSM (51.3%) compared to JXJJ (43.0%), indicating better maintained PSII–PSI co-ordination in JXJJ under drought stress. (I_1_ − D_2_)/D_2_ of both provenances were significantly affected by drought stress. DF measurements showed significantly greater disruption of PSII energy transfer in FJSM after 20 d of drought, with the (I_1_ − D_2_)/D_2_ ratio decreasing by 64.0% compared to only 57.5% in JXJJ.

## 3. Discussion

The results of this study revealed significant provenance-based differences in the physiological responses of *C. lanceolata* seedlings to drought stress. These findings highlight the importance of provenance-specific traits in determining drought tolerance, as evidenced by variations in gas exchange, chlorophyll fluorescence, and related parameters between JXJJ and FJSM. The difference between FJSM and JXJJ may be due to the combined influence of genetic basis and environmental factors. On the one hand, they may have a different genetic basis. However, their genetic basis is still unclear, and the relevant regulatory genes and molecular mechanisms need to be further studied. On the other, due to the different latitudes, there are significant differences in climatic conditions such as temperature and precipitation for their growth. Since two-year-old seedlings were obtained, the environment may have already exerted a significant influence on them.

Forest soil is a vital component of forest ecosystems [19], and SMC is a critical factor affecting plant growth and development. Its fluctuations under drought stress are particularly noteworthy [20]. Previous research has highlighted that physiological characteristics can vary notably among different plant species and cultivars [21,22]. The intensification of drought stress significantly affects SMC, directly influencing the photosynthesis responses of *C. lanceolata*. In this study, there was marginal difference in SMC, indicating that both provenances experienced similar impacts from drought stress (Figure 1A). The progressive decline in SMC during the drought stress period significantly affected gas exchange parameters in both provenances. Most current studies on plant gas exchange focus on parameters such as Pn, Gs, and transpiration rate (Tr), along with internal and external factors that influence photosynthesis [23]. It has been demonstrated that leaf gas exchange parameters are more sensitive to leaf water content than to SMC [24]. We observed variations in gas exchange parameters between JXJJ and FJSM, with Pn showing significant differences after 10 d of drought stress, while Gs and Ci exhibited significant divergence at later time points, indicating better maintenance of photosynthetic activity and water-use efficiency under drought conditions (Figure 1B–D). The inhibition of photosynthesis under drought stress can be attributed to two factors, stomatal and non-stomatal limitations. Under mild drought stress, the decline in Pn resulted from a decrease in Ci and Gs is primarily due to stomatal limitations. In contrast, under severe drought stress, the inconsistent trends in Ci and Gs changes result in a decline in Pn attributed to non-stomatal limitations [25]. The results of this experiment showed that, under drought stress, the Pn and Gs of *C. lanceolata* seedlings decreased, while Ci increased (Figure 1B–D), indicating that the reduction in Pn was caused by non-stomatal limitations.

We investigated the changes in the photosynthetic electron transport chain of *C. lanceolata* seedlings under drought stress by simultaneously measuring PF, DF, and MR, as well as the variation characteristics of these parameters. The PF dynamics, characterized by the rise from the minimum O point to the maximum P point, primarily reflect changes in the initial photochemical activity of PSII [26]. The OJIP transients showed substantial differences between the two provenances under drought stress. With the aggravation of drought stress, the value of F_P_ decreased, the amplitude of I-P phase decreased, the amplitude of J-step increased (Figure 2). JXJJ exhibited relatively stable OJIP curves with slight changes in the fluorescence rise at the J, I, and P steps (Figure 2A). The decrease in the F_P_ might be caused by a reduction in the number of PSII reaction centers, increased non-radiative dissipation of energy by PSII antenna pigments, and impairment at the PSI acceptor side [27,28,29]. Meanwhile, the rise in the F_J_ is attributed to reduced electron transfer efficiency at Q_A_ [30], while the suppression of I-P phase amplitude indicates that drought affects changes in the redox state of PSI [31]. The apparent J-step prominence in normalized traces reflects reduced electron transport beyond QA^−^ (J-I-P segment), consistent with lower Ψ_O_ and δR_O_ values (Figure 2B,E). In the course of this study, the positive K-band and L-band were induced by severe drought stress (Figure S1). Similarly, a positive K-band and/or a positive L-band caused by drought stress have also been observed in previous studies [32]. The pronounced K-band indicated that drought stress may disrupt the oxygen-evolving complex (OEC) on the donor side of PSII, impairing its electron transport capacity. The positive L-band indicated a reduction in connectivity among PSII units [27,31]. This decreased connectivity leads to the lower excitation energy utilization efficiency and the reduced stability of the PSII units [33,34]. Since the OEC participates in the photolysis of water during photosynthesis, our findings indicated that drought stress alters the process of water oxidation. Under drought stress, there is no significant difference in the K-band between FJSM and JXJJ, while the L-band of FJSM is more pronounced than JXJJ (Figure S1A–D), suggesting that the OEC in both FJSM and JXJJ suffered similar damage. Simultaneously, a clearer positive L-band was observed in FJSM (Figure S1E–H). Higher positive L-band indicates a greater reduction in connectivity among PSII units. Lower connectivity leads to decreased energy efficiency and reduced stability of PSII units [35].

A JIP-test, based on OJIP transients, has been developed to reveal detailed information about each step of the photosynthetic process [36]. In the current study, analysis of JIP-test parameters further confirmed the contrasting responses of the two provenances (Figure 3). ABS/RC reflects the ratio of the molecular weight of chlorophyll a in the PSII antenna complex to the active RCs [37]. As some active RCs were inactivated under the drought treatment, ABS/RC and DI_O_/RC were increased. This observation reflected elevated energy dissipation per RC and reduced photochemical efficiency. Simultaneously, a decrease in F_V_/F_M_ and PI_ABS_ indicated severe damage to PSII reaction centers and diminished overall photosynthetic performance. When exposed to drought stress, the ψ_O_ and δR_O_ values from both provenances exhibited varying degrees of decline. The significant reductions in φE_O_ and ψ_O_ indicate that photosynthetic electron transport was inhibited beyond Q_A_ [38], consistent with the relatively higher J-step levels observed in the V_t_ curves of the OJIP transient. The lower δR_O_ levels suggest a reduction in electron flow from the PSI acceptor side, likely due to the inactivation of ferredoxin-NADP^+^ reductase (FNR) [28]. JXJJ, on the other hand, demonstrated relatively stable JIP-test parameters, highlighting its superior ability to maintain electron transport efficiency and photochemical activity under drought stress.

The MR signal serves as an excellent complementary technique to PF measurements, offering additional insights into photosynthetic electron transport processes [39]. A typical 820 nm kinetic curve consists of a rapidly decreasing phase followed by a slowly increasing phase [40]. The MR/MR_O_ values, reflecting the redox state of P700 and PC, showed significant changes in both provenances under drought conditions (Figure 4A,B). The reduction rate of PC and PSI is equivalent to their oxidation rate at the lowest point of the decline phase [41]. The lowest point of the fast decrease phase of the MR/MR_O_ transient appeared at a later time accentuated with the intensification of drought stress. JXJJ maintained higher MR/MR_O_ values throughout the drought stress period (Figure 4A), indicating the better preservation of PSI redox balance. Conversely, FJSM exhibited a steeper decline in MR/MR_O_, particularly after 10 d of stress treatment (Figure 4B), suggesting more severe disruption in PSI electron transport due to drought-induced oxidative stress. As drought stress progressed, V_ox_ exhibited a consistent increase in both JXJJ and FJSM seedlings (Figure 4C). This indicated that an enhanced oxidation of P700, likely due to a reduction in electron donation from PSII to PSI. However, the increase in V_ox_ was more pronounced in FJSM, especially after 15 and 20 d of drought (Figure 4C). It suggested that PSI in FJSM was more affected by the impaired electron transport chain, leading to a higher accumulation of oxidized P700 [42,43]. Alternatively, V_red_ showed a marked decline in both provenances over the drought period, with FJSM experiencing a sharper decrease compared to JXJJ (Figure 4D). Previous studies have reported a reduction in the rate of P700 reduction, which may be attributed to damage or limitations on the acceptor side of PSI under drought stress [44,45]. The lower V_red_ values in FJSM suggested that drought-induced disruptions in PSI electron acceptors, such as ferredoxin or NADP^+^ reductase, were more severe in this provenance.

The DF signal provides additional information about changes in the photosynthetic electron transport process [46,47,48]. The dynamic curves of DF intensity for both JXJJ and FJSM demonstrate significant changes over the course of drought stress (Figure 5A,B). The parameters I_1_, I_2_, and D_2_ provide insights into the energy transfer and the functional integrity of PSII. The I_2_/I_1_ ratio, shown in Figure 5C, increased progressively during the drought stress, indicating alterations in the ETC. After 20 d of drought treatment, FJSM exhibited a significantly higher I_2_/I_1_ compared to JXJJ, which suggested that the electron flow through PSII in FJSM was more severely disrupted under prolonged stress. The higher I_2_/I_1_ in FJSM may reflect impaired energy dissipation mechanisms or reduced efficiency of energy transfer between PSII reaction centers, leading to increased fluorescence intensity [49]. Conversely, the (I_1_ − D_2_)/D_2_ ratio (Figure 5D) decreased with the progression of drought, highlighting a decline in the capacity for charge recombination within PSII. JXJJ exhibited a significantly higher (I_1_ − D_2_)/D_2_ ratio than FJSM after 20 d of drought, indicating better PSII stability under prolonged stress. Above results indicated that JXJJ retained better PSII efficiency and stability under stress conditions [50,51].

## 4. Materials and Methods

### 4.1. Plant Material and Treatment

The experiment was conducted in artificial climate champers of Jiyang College of Zhejiang A & F University (29°44′51″ N, 120°15′17″ E), Zhuji, Zhejiang Province, China. The experimental conditions were maintained at a temperature of 25 °C/20 °C (day/night), with a relative humidity of 70% ± 5%. The photoperiod was set to 16 h/8 h (day/night), with a light intensity of 600 μmol m^−2^ s^−1^. In this experiment, two-year-old *C. lanceolata* seedlings were obtained from Sanming, Fujian Province (FJSM; 116°48′52″ N, 26°10′17″ E) and Jiujiang, Jiangxi Province (JXJJ; 115°54′40″ N, 29°36′30″ E). Plants were cultivated in a pot with a diameter of 16 cm and a depth of 24 cm, containing 6 kg of loam soil (pH = 4.7). Seedlings were watered continuously for two weeks to maintain the substrate moisture content at 70–80% of the maximum field water capacity, thus ensuring the normal growth of the seedlings. Subsequently, a natural drought stress treatment was initiated by ceasing water supplementation. Measurements were taken at days 0 (control), 5, 10, 15, and 20 days (d) after water cessation. The experiment included six biological replicates, with each replicate consisting of six seedlings.

### 4.2. Soil Moisture Content and Gas Exchange Measurement

Soil moisture content (SMC) was measured using a soil temperature and humidity sensor (HSTL-TRSC02, Beijing, China) at 8 am each day. Measurements were taken three times per pot, and the average value was calculated. Gas exchange parameters of the leaves were measured using a LI-6400XT portable photosynthesis system (LI-COR, LI-COR, Lincoln, NE,USA). Measurements were conducted on a sunny day, from 9:00 to 12:00 [52]. Small steel cylinders filled with carbon dioxide were used to ensure the stabilization of gas concentrations. For each treatment, three leaves were selected from similar positions on the main stem. The measurements utilized the built-in red-blue light source set at 1200 μmol∙m^−2^∙s^−1^, a CO_2_ concentration of 400 μmol∙mol^−1^, a leaf chamber temperature of 25 °C, and a flow rate of 300 mmol∙s^−1^. The system automatically recorded physiological parameters, including net photosynthetic rate (Pn), stomatal conductance (Gs), and intercellular CO_2_ concentration (Ci).

### 4.3. Simultaneous Measurements of the Kinetics of PF, DF, and MR

The kinetics of PF, DF, and MR were simultaneously measured by the Multifunctional Plant Efficiency Analyzer M-PEA (Hansatech, Norfolk, UK) at room temperature (25 °C). All leaves were dark-adapted for 30 min before measurements. The M-PEA emits light at wavelengths of 627 ± 10 nm for photochemical light, 820 ± 25 nm for modulated light, and 735 ± 15 nm for far-red light [41]. The intensity of the saturating light pulse was set to 5000 μmol photons m^−2^ s^−1^. PF, DF, and MR signals were recorded using alternating light and dark intervals. PF and MR measurements were taken during the light intervals when the actinic light was on, while DF was recorded during the dark intervals when the light was off [53]. The parameters of the JIP-test connect various steps of the photosynthetic process and describe the efficiency of electron transport along the PSII redox state, progressing through the electron transport chain to the ultimate electron acceptor in PSI. The OJIP transient rise was analyzed using the JIP test for detailed assessment of chlorophyll fluorescence dynamics (Table S1).

### 4.4. MR and DF Analysis

MR provides insights into electron transport dynamics between the plastoquinone (PQ) pool and the electron acceptors in PSI, reflecting changes in the redox state of P700 and plastocyanin (PC) [54]. MR is represented as the ratio MR/MR_0_, where MR_0_ corresponds to the initial value at the onset of actinic illumination (recorded at 0.7 ms, the first reliable measurement), and MR denotes the modulated reflection signal captured during the illumination period. To assess PSI activity, the absolute value of the maximum decrease in the slope (V_ox_, within the 0.7–3 ms range) of MR/MR_0_ is calculated. The absolute value of the initial rate of P700^+^ re-reduction (V_red_) is then used to evaluate the activity of cyclic electron transport (CET) [55].

DF is primarily emitted from PSII and its decay follows a polyphasic function over various time domains, spanning from microseconds to minutes [46]. A specific dark time point of 20 ms was selected for constructing DF induction kinetics. These kinetics are assumed to be influenced by the redox state of the PSII primary electron acceptor.

### 4.5. Statistical Analysis

All data were obtained from three biological replicates and are presented as mean ± standard error. Repeated measures ANOVA (RM-ANOVA) was performed using SPSS v20.0 (IBM, Armonk, NY, USA), and statistical significance was set at *p* < 0.05. Data visualization was carried out using Origin v9.8.0 (Origin Lab, Northampton, MA, USA).

## 5. Conclusions

In summary, the superior drought tolerance of JXJJ can be attributed to its more efficient stomatal regulation, enhanced photochemical efficiency, and reduced oxidative damage under water-limiting conditions. This study underscores the significant role of both stomatal and non-stomatal factors in shaping the drought response of *C. lanceolate*, and offers valuable insights for improving drought resistance in forest species. Future research should focus on identifying the molecular mechanisms driving drought tolerance and exploring genetic strategies to enhance drought resilience in forestry applications.

## Data Availability

The datasets generated during and/or analysed during the current study are available from the corresponding author on reasonable request.

## Supplementary Materials

The following supporting information can be downloaded at: https://www.mdpi.com/article/10.3390/plants14101487/s1, Table S1: Summary of JIP-test parameters and formulae; Figure S1: Effect of drought treatment on the shape of the K-band and L-band.
