# Supplementary material for: Drought-Driven Divergence in Photosynthetic Performance Between Two Cunninghamia lanceolata Provenances: Insights from Gas Exchange and Chlorophyll Fluorescence Dynamics
Source: Plants (Basel). 2025 May 15;14(10):1487. doi: 10.3390/plants14101487 (PMC12115317; doi:10.3390/plants14101487)
Supplement: Supplementary file 1 [file plants-14-01487-s001.zip › plants-3612969-supplementary.pdf]

**Table S1.** Summary of JIP-test parameters and formulae.

| | |
|---|---|
| **Measured parameters from the OJIP transient** | |
| $F_O$ | Minimum reliable fluorescence intensity (20 μs) |
| $F_J$ | Fluorescence at the J-step (2 ms) |
| $F_I$ | Fluorescence at the I-step (30 ms) |
| $F_P$ | Maximum fluorescence intensity at the P step |
| **Specific energy flux and phenomenological flux** | |
| $ABS/RC= [M_O/V_J][1/(F_V/F_M)]$ | The absorption of antenna chlorophyll per PSII reaction center |
| $DI_O/RC=(ABS/RC)-(TR_O/RC)$ | Dissipation per Reaction Center |
| $ET_O/CS_M=(F_M-F_O)\times(1-V_J)\times(RC/CS_M)$ | Electron transport rate per unit area |
| $TR_O/CS_M=(F_M-F_O)\times(RC/CS_M)$ | The rate of primary photochemical capture per unit cross-sectional area ($CS_m$) |
| **Quantum yields and performance index** | |
| $F_V/F_M=(F_M-F_O)/F_M$ | The maximum photochemical efficiency of PSII |
| $\varphi D_O=F_O/F_M$ | The quantum efficiency of energy dissipation |
| $\varphi E_O=[1-(F_O/F_M)](1-V_J)$ | The quantum yield for electron transport |
| $\Psi_O=(1-V_J)/(1-V_I)$ | The probability that an electron moves further than $Q_A^-$ |
| $\delta R_O=(1-V_I)/(1-V_P)$ | The efficiency of an electron beyond $Q_A^-$ that reduced PSI acceptors |
| $PI_{ABS}=$ （$RC/ABS\times\varphi P_O\times\Psi_O)/(1-\varphi P_O)$ | The performance index on an absorption basis |

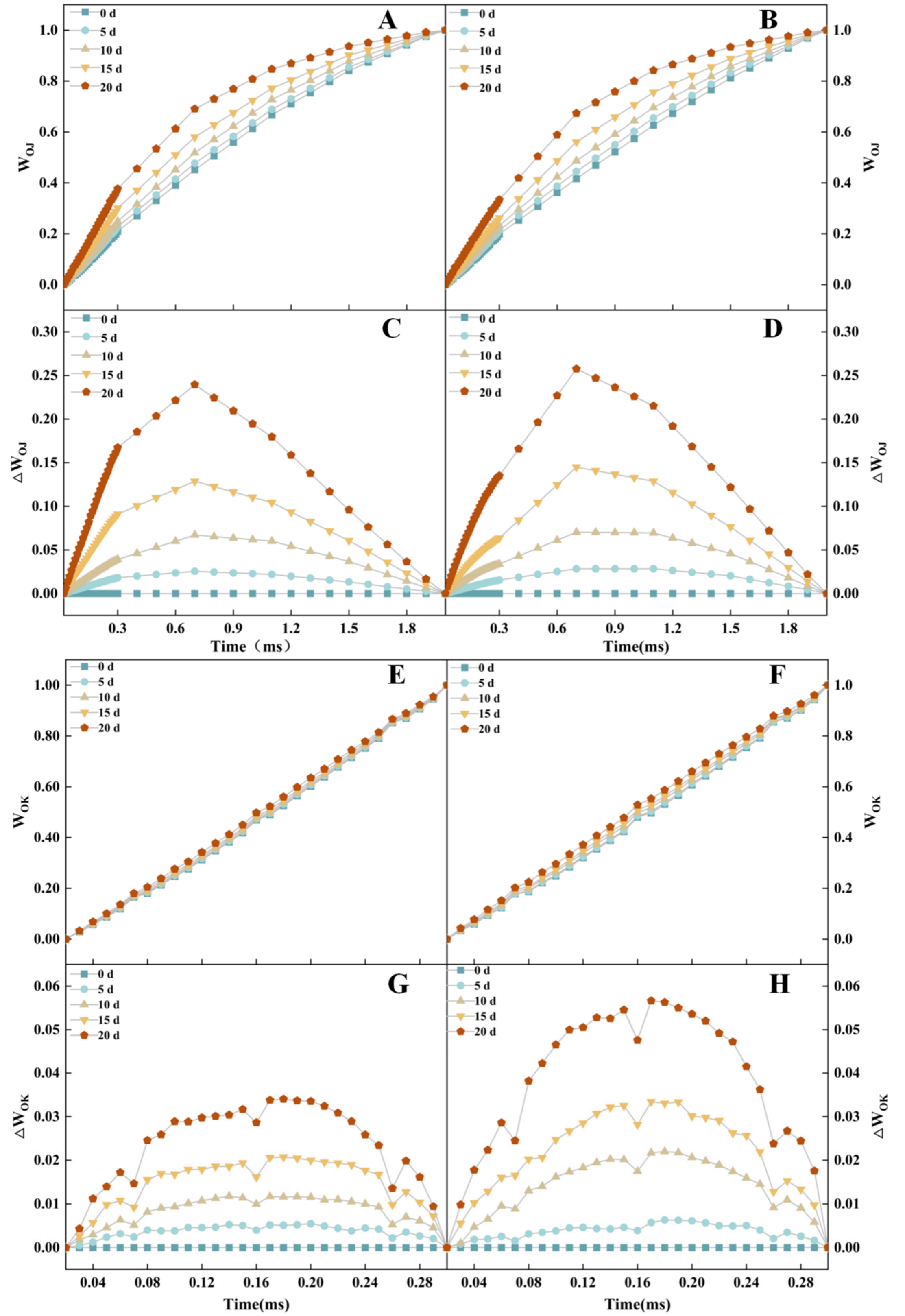


Figure S1. Effect of drought treatment on the shape of the K-band and L-band. (A-D) The PF transient curves normalized between $F_O$ and $F_J$, and their difference kinetics (K-band). (E-H) The PF transient curves normalized between $F_O$ and $F_K$, and their difference kinetics (L-band). Note: the left panels indicate JXJJ, the right panels indicate FJSM. Data are the mean of six biological replicates.
